# Using COVID‐19 as a teaching tool in a time of remote learning: A workflow for bioinformatic approaches to identifying candidates for therapeutic and vaccine development

**DOI:** 10.1002/bmb.21413

**Published:** 2020-07-29

**Authors:** Samantha Bryce, Kevin N. Heath, Luca Issi, Elizabeth F. Ryder, Reeta P. Rao

**Affiliations:** ^1^ Biology and Biotechnology Department Worcester Polytechnic Institute Worcester Massachusetts USA; ^2^ Bioinformatics and Computational Biology Program Worcester Polytechnic Institute Worcester Massachusetts USA

**Keywords:** active learning, cellular biology, computational biology, distance learning, immunology, inquiry‐based teaching, integration of research into undergraduate teaching, molecular biology, virology

## Abstract

The COVID‐19 pandemic has led to an urgent need for engaging computational alternatives to traditional laboratory exercises. Here we introduce a customizable and flexible workflow, designed with the SARS CoV‐2 virus that causes COVID‐19 in mind, as a means of reinforcing fundamental biology concepts using bioinformatics approaches. This workflow is accessible to a wide range of students in life science majors regardless of their prior bioinformatics knowledge, and all software is freely available, thus eliminating potential cost barriers. Using the workflow can thus provide a diverse group of students the opportunity to conduct inquiry‐driven research. Here we demonstrate the utility of this workflow and outline the logical steps involved in the identification of therapeutic or vaccine targets against SARS CoV‐2. We also provide an example of how the workflow may be adapted to other infectious microbes. Overall, our workflow anchors student understanding of viral biology and genomics and allows students to develop valuable bioinformatics expertise as well as to hone critical thinking and problem‐solving skills, while also creating an opportunity to better understand emerging information surrounding the COVID‐19 pandemic.

## 1 INTRODUCTION

Since the start of the coronavirus pandemic of 2019 (COVID‐19; etiological agent SARS CoV‐2), educators have had to rapidly change how they teach concepts and techniques to university students, in part because they have lost the ability to hold face‐to‐face classes or provide hands‐on laboratory experiences. The effectiveness of an inquiry‐based approach to laboratory course work is well established,^1^, ^2^ but it is difficult to replicate in remote learning. While studies indicate that student comprehension and grades are comparable to physical laboratories when using computer‐simulated experiments,^3^, ^4^ data also suggest that students do not prefer the exclusive use of simulations^3^ as a learning tool. This report provides an inquiry‐based digital alternative, leveraging publicly available genomic data to anchor student learning about a pandemic with evolving information. In a time of uncertainty, we present a unique opportunity to utilize alternative methods of digital instruction while also strengthening students' understanding of COVID‐19 and giving them the critical thinking skills necessary for original research.

**FIGURE 1:**
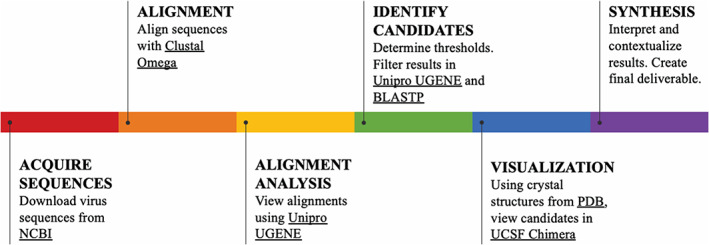
General workflow of student learning and bioinformatics tools used to align, analyze, and visual conserved regions among proteins

Here we introduce a bioinformatics workflow that promotes original research and flexible thinking, which is designed to be accessible to students of all bioinformatics levels (Figure 1, Appendix S1). In the context of COVID‐19, the workflow motivates students to analyze viral genomes, identify the similarities and differences between genetically related corona viruses that have also caused sizeable outbreaks in humans, and learn how to leverage that understanding to identify targets that might be useful for vaccines or therapeutics. The connection to this real‐world situation will likely lead to greater interest from students,^5^ and allow them to critically evaluate the current events.

Special attention was paid to accessibility during the development of this learning module. All software, databases, and servers used are in the public domain, and therefore accessible to all students, instructors, and institutions free of charge. This approach eliminates concerns regarding university computing capacity and software subscriptions, as well as students' ability to afford software. The workflow requires only an internet connection and a personal computer. Finally, all steps in the process utilize resources that are user friendly, with programs selected to be approachable to students and instructors with little prior experience in bioinformatics. Tutorial resources are available for all software, giving students guidance even when working independently. The workflow also serves to introduce biology students to diverse bioinformatics tools using a highly motivating example.

This flexible computationally based module is meant as a starting point with several opportunities for customization, some of which are outlined later. The workflow strives to make the current pandemic accessible to students by effectively utilizing remote project‐based learning yielding original research. While the workflow was created and tested for use with coronavirus strains, it is important to note that the methods described could be applied to other pathogens of interest to a particular course (Appendix S2) and can be adapted as necessary to suit instructor needs. Ultimately, this workflow is intended to be used as either an alternative to a sequence of traditional laboratory sessions, or as a standalone project‐based approach for biology students to learn bioinformatics skills.

## 2 NECESSARY PREPARATION AND TIME COMMITMENT

Our workflow is primarily designed for students with some previous coursework in cellular and molecular biology, but minimal bioinformatics experience. We assume a basic understanding of BLAST and multiple alignments, which can be supplemented with publicly available tutorials^6^ if necessary. The workflow is appropriate for both more advanced undergraduate and graduate life sciences students, and different levels of support can be provided to students to adjust for experience level. We suggest that students complete the project in small groups of two to three people. The exercise can be conveniently divided into three parts, which can either be completed entirely during class sessions or introduced during class and finished independently: (i) identify sequences for comparisons and perform alignments, (ii) analyze alignments including setting thresholds and identifying candidates that meet said thresholds, (iii) visualize candidates and perform literature review to determine appropriateness of candidates. The time needed for each part will vary based on previous experience, as well as on the amount of supporting information students are given. We recommend allotting ~2 h for each part if scaffolding (Appendix S1) and sequences are provided. For more independent investigations, we recommend ~5 h per part, giving students ample time for research on the virus, learning to use the software tools, and experimenting with different parameter settings.

## 3 WORKFLOW

The goal of this workflow (Figure 1) is to identify small well‐conserved regions of a protein that can serve as targets for drugs and/or vaccines. Genetic regions are conserved through evolution because they offer a fitness advantage. For a virus, such peptides are likely important for transmission and/or virulence. These conserved regions are less likely to mutate, because they are likely vital to the viral life cycle,^7^ and therefore present avenues for drug or vaccine development with long lasting efficacy. Conserved regions of proteins on the outer surface of the virus may be good targets for vaccine development, because they are accessible to the immune system.^8^ However, such targets may prove problematic, because mutation of viral surface proteins is a mechanism by which viruses evade host immune surveillance.^9^ On the other hand, conserved regions of proteins in the core of the virus may serve as targets for antiviral therapy.

The workflow begins with students identifying organisms that are appropriate to align, such as viruses of the same genus (e.g., coronavirus) that are known to cause serious disease in humans. Students could identify these sequences themselves, or they could be provided by the instructor. In addition, the instructor could choose to provide background information about the virus of interest in the form of a lecture. Alternatively, students may be assigned an independent study to review the viral life cycle, and then identify important viral proteins. In the case of SARS CoV‐2, these would include the spike protein on the viral surface (which is important for initial infection)^10^ or the RNA‐dependent RNA‐polymerase (which is not present in the human host and thus could be a good therapeutic target).

Students then export protein sequences from the selected genomes from the National Center for Biotechnology Information (NCBI) database^11^ and align them using Clustal Omega.^12^ In the case where the selected strains have many proteins of interest, all amino acid sequences of each strain can be concatenated in a text file and then aligned to achieve a general sense of which proteins are most conserved. Individual proteins of interest should subsequently be realigned and analyzed independently of the other proteins. Alignments are visualized and analyzed using Unipro UGENE.^13^ Students set their own thresholds for region length and percentage conservation, and then filter results in Unipro UGENE to identify candidates meeting these criteria. Thresholds should be informed by both the literature^14^ and preliminary analysis of the alignments to identify filtering that is suitable to project goals while also accounting for alignment length. In general, longer alignments with higher percentage conservation are less likely to occur by chance, and also produce fewer spurious matches to human sequences. Reasonable initial parameter values include peptide lengths ≥20 amino acids, with conservation identity ≥50%.

Peptide candidates are then evaluated for autoantigenicity by examining their similarity to human proteins. Strong similarity with human proteins would indicate a greater likelihood that a vaccine targeting this peptide would likely have undesirable side effects, such as auto immunity.^14^ Similarly, an antiviral agent to such a peptide could have toxic side effects. The NCBI BLASTP^15^ tool is used to search for the candidate peptide sequence in human proteins. BLASTP generates an *E* value, which is the number of anticipated matches identified by chance; this value is scaled by the size of the database and the length of the query sequence. When a viral peptide is compared against the human proteome, a small E value indicates a closer match, and thus a higher probability of autoimmune responses or toxic side effects. Although there are no arbitrary cutoffs, *E* values smaller than 0.001 typically indicate true matches. As a cautious rule of thumb, we suggest that students try to identify peptides with alignments against the human genome having *E* values >0.1.

Once peptide candidates are identified, the proteins they are derived from are located by cross‐referencing sequences in the RCSB Protein Data Bank (PDB).^16^ Proteins containing the best candidate peptides can then be visualized in UCSF Chimera,^17^ with regions of interest highlighted and/or labeled. This visualization tool reinforces a critical biochemical concept of protein structure–function relationships. Specifically, such 3D rendering aids visualization of regions that are buried in the protein therefore inaccessible to drugs or antibodies. In order to utilize this part of the workflow, students should choose proteins whose crystal structures are available; de novo calculation of 3D structure is outside the scope of this exercise.

The final step of the pipeline includes a deliverable, which could be a laboratory report, presentation, or more formal paper. Requiring a final product ensures that students reflect upon the outcome of their work as a whole, rather than just completing a series of steps using software tools.

## 4 SAMPLE RESULTS: COVID‐19

To ensure the efficacy and ease of this workflow, we tested our methods using seven CoVs that have caused serious disease in humans but vary with respect to their epidemiology.^18^ The goal was to employ the workflow to identify well‐conserved regions that could be further investigated as targets for vaccine or drug development. This workflow was tested over the course of ~2 weeks in the spring of 2020 by two graduate students: one biologist with more knowledge of molecular biology but minimal prior bioinformatics experience and one computational biologist with more familiarity with the programs used. Here we include some of our results to show what a deliverable from a student might look like in practice. These results demonstrate the flexibility of our workflow and its appropriateness for students of diverse biology and bioinformatics experience levels.

Through a literature search, we identified seven CoVs that have been known to cause serious disease in humans.^18^ COVID‐19 is the most recent of the CoV diseases, with SARS CoV‐2 as the causative agent responsible.^19^ To perform the analysis, we exported these viral CoV genomes from NCBI: HCoV‐229E (MF542265.1), HCoV‐HKUA1 (AY884001.1), HCoV‐NL63 (JX104161.1), HCoV‐OC43 (AY391777.1), MERS‐CoV (JX869059.2), SARS‐CoV (AY274119.3), and SARS‐CoV‐2 (MN908947.3). We first analyzed all of the proteins by performing an alignment of the concatenated proteins. Then based on preliminary analysis we subsequently focused on the two overlapping viral polypeptides, which together constitute most of the translated regions of the virus; sequences were aligned using Clustal Omega. Putative peptides with ≥20 amino acids (AA) with ≥50% conservation were identified and analyzed using Unipro UGENE. These thresholds were set based on the conservation of the alignments while balancing the desire for unique conserved targets.

Based on the initial parameters set (≥20 AA, ≥50% conservation), eight highly conserved amino acid sequences were first identified (Table 1). Using the RCSB Protein Data Bank, all eight candidates were identified as part of the viral replicase polyproteins PP1a and PP1ab, which are subsequently cleaved into 16 nonstructural proteins (nsp).^20^ All candidates except for #2 (Table 1) met the *E* value threshold (>0.1) when compared with the human proteome using BLASTP. This suggests that the human proteome does not contain close matches to these peptides, which therefore present reasonable targets for disease intervention strategies. To better understand some of the most compelling candidates, the peptides were visualized in the context of 3D crystal structure of the protein using UCSF Chimera software.

**TABLE 1:** Amino acid (AA) sequence in SARS‐CoV‐2 of the eight highly conserved candidates. Candidates are organized in this table based on their sequential position

#	Locus	Protein function	AA sequence in SARS‐CoV‐2	Length
1	nsp12	RNA‐dependent RNA polymerase^20^	GIVGVLTLDNQDLNGNWYDFGDFI	24
2			YVKPGGTSSGDATTAYANSVFNICQAVTANV	31
3			KHFSMMILSDDAVVCFNSTYA	21
4			VLYYQNNVFMSEAKCWTETDLTKGPHEFCSQHTMLVK	37
5	nsp13	RNA helicase^20^	QGPPGTGKSHFAIGLALYYPSARIVYTACSHAAVDALCE KA	41
6			KAVFISPYNSQNAVASKILGLPTQTVDSSQGSEYDYVIF TQTTETAHSCNVNRFNVAITRAKVGILCIMSD	71
7	nsp16	Methyltransferase^21^	LPKGIMMNVAKYTQLCQYLNTLTLAVPYNMRVIHFGA GSDKGVAPGTAVLRQWLPTGTLLVDSDLNDFVSDAD	73
8			KLALGGSVAIKITEHSWNADLYKLMGHFAWWTAFVTN VNASSSEAFLIGCNYLGK	55

**FIGURE 2:**
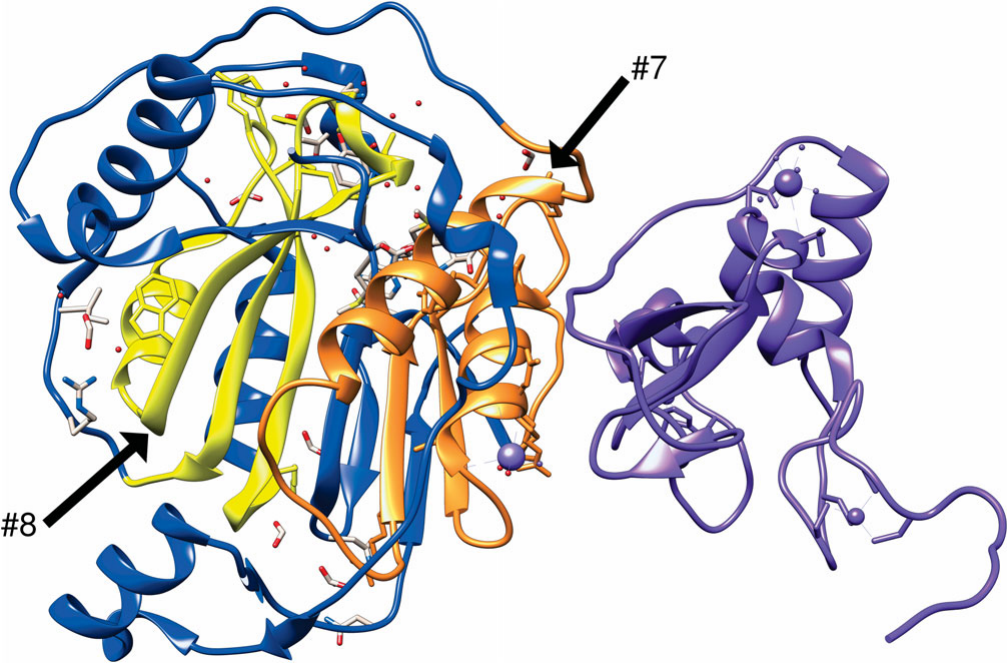
Visualization of the nsp16‐nsp10 complex with candidates #7 and #8 identified. The nsp16 protein is denoted and blue, the nsp10 protein is denoted in purple, and candidates are denoted in yellow and orange. Labeled structure was generated in UCSF Chimera

All peptides (Table 1) mapped to proteins in the core of the virus. Therefore, they are better suited for antiviral drug development rather than vaccines, which typically target proteins on the surface of the virus. We further investigated two of the longest and best conserved peptides (Table 1) and mapped them to nsp16. The nsp16 protein is a methyltransferase that is activated by its interaction with nsp10.^21^ In the absence of nsp10, nsp16 has no enzymatic activity,^22^ underscoring the importance of protein–protein interactions in biology. Since the structures of nsp16 and nsp10 are available, we generated a visualization showing the precise location of the candidate peptides within the nsp16–nsp10 complex (Figure 2). We noted that the target peptides (#7 and #8, Table 1) are on exposed regions of the protein, suggesting these regions would be accessible to antiviral drugs. Further survey of the literature suggested that the nsp 16–nsp 10 complex is involved in viral replication and pathogenesis^23^; therefore, functional inhibition should inactivate the virus.^24^ More recently, the complex has been identified as a potential avenue for antiviral development, with particularly compelling hope for its eventual efficacy, given how well it is conserved among coronaviruses.^25^

## 5 POTENTIAL PITFALLS

An alignment itself may not yield interesting candidates, especially if the sequences are genetically divergent and/or not enough sequences are analyzed; we encourage using 5+ strains to minimize these possibilities. If students are struggling to identify candidates in UGENE, they should first look at the conservation map at the bottom of the screen (Appendix S1), and then move to the area/s with the highest peaks. Students can be encouraged to try different thresholds for both length and percentage identity, which may yield more candidates. Making such changes has the potential to also alter the outcomes of the analysis—for example, a shorter length or lower conservation identity may result in peptide candidates with *E* values that when compared with human proteins are indicative of a poor vaccine target. In anticipation of technical difficulties, we provide a detailed protocol including screenshots, which can be used as a guide for those with minimal bioinformatics background (Appendix S1).

## 6 DIFFERENTIATION

This workflow can be customized to meet different learning outcomes, for the course as well as the student, by adapting both the rigor and emphasis of the project. For example, students with less bioinformatics experience could be provided with our detailed protocol (Appendix S1) and be allowed more time to spend reviewing software tutorials, while students with more advanced computational skills could amplify the workflow by expanding upon their alignment analysis with other programs such as Jalview 2.^26^ Similarly, students with less biological knowledge could be provided with a list of viral accession numbers to begin with, while those with more background could find these themselves from the literature.

More broadly, both the emphasis and the final product (an assignment or presentation) can be adjusted. In the example above, we briefly discuss the function of a protein complex with a well conserved region among coronaviruses with the goal of identifying potential targets for drug/vaccine development. The COVID‐19 example presented here is better suited for students with background in virology, and can be further guided by first having students read popular press^27^ and/or relevant reviews as primers on the topic,^28^, ^29^ depending on students' prior knowledge. However, we demonstrate the versatility of the workflow (Appendix S2) for other infectious microbes. Other end points for the module could include phylogenetic analysis of conserved proteins for genetics laboratories, or more thorough investigation and interpretation of shared conservation with human proteins in the context of autoimmune responses for immunology laboratories. These examples represent just some of the ways that the workflow can be differentiated to meet instructor needs.

## 7 ADDITIONAL LEARNING OPPORTUNITIES FOR INSTRUCTOR REINFORCEMENT

The workflow can be further differentiated to create additional learning opportunities for students. Examples of concepts that can be reinforced by the instructor: (i) distinguish between vaccines and therapeutics; (ii) emphasize how surface proteins are recognized by the immune system and are therefore good targets for vaccine development; (iii) pose cross‐reactivity with auto antibodies as a significant barrier to vaccine development; (iv) highlight that changes in surface proteins pose a significant challenge for vaccine development (e.g., HIV); (v) differentiate why certain vaccines provide limited immunity and requiring repeated vaccination (Flu); (vi) extrapolate the implications for testing active disease or acquired immunity; (vii) elaborate on the COVID‐19 disease manifestation and how it relates to a hyperactivity of the immune system (cytokine storm).

## 8 CONCLUSIONS

The learning module outlined in this paper leverages project‐based learning methodologies and provides a rigorous alternative to laboratory projects that can be administered remotely. Students have the opportunity to perform original data analysis using accessible tools and databases, while practicing critical thinking. We demonstrated the flexibility of our workflow, which allows for customization to meet both instructor and student needs. The workflow, supplemented with materials provided in the appendix, provides the framework necessary to guide instructors and students. Ultimately, it allows students to hone their problem‐solving skills while also giving them a means of understanding current events.

## Supporting information


**DATA S1** Supplemental materials 1 and 2

## References

[1] Suits J . Assessing investigative skill development in inquiry‐based and traditional college science laboratory courses. Sch Sci Math. 2010;104:248–57.

[2] Parappilly M , Siddiqui S , Zadnik M , Shapter J , Schmidt An L . Inquiry‐based approach to laboratory experiences: investigating students' ways of active learning. Int J Innov Sci Math Educ Former CAL‐Lab Int. 2013;21 42–53.

[3] Wiesner T , Lan W . Comparison of student learning in physical and simulated unit operations experiments. J Eng Educ. 2004;93:195–204.

[4] Reece AJ , Butler MB . Virtually the same: a comparison of stem students’ content knowledge, course performance, and motivation to learn in virtual and face‐to‐face introductory biology laboratories. Research and teaching. J Coll Sci Tech. 2017;46:83–9.

[5] Howard RE , Boone WJ . What influences students to enjoy introductory science laboratories? Some keys to building a Student's enjoyment of the introductory science laboratory. J Coll Sci Teach. 1997;26:383–7.

[6] D. Wheeler , M. Bhagwat (2007) BLAST QuickStart. Humana Press. PMC4780883 17993672

[7] Khan AM , Miotto O , Nascimento EJM , Srinivasan KN , Heiny AT , Zhang GL , et al. Conservation and variability of dengue virus proteins: Implications for vaccine design. PLoS Negl Trop Dis. 2008;2:e272. 18698358 10.1371/journal.pntd.0000272 PMC2491585

[8] Leifert JA , Whitton JL . In: Ertl HCJ, editor. DNA Vaccines. Philadelphia, PA: Springer Science & Business Media; 2003.

[9] Charles J , Janeway A , Travers P , Walport M , Shlomchik MJ . Pathogens have evolved various means of evading or subverting normal host defenses Immunobiology. The immune system in health and disease. 5th ed. New York: Garland Science; 2001.

[10] Cheng VCC , Lau SKP , Woo PCY , Yuen KY . Severe acute respiratory syndrome coronavirus as an agent of emerging and reemerging infection. Clin Microbiol Rev. 2007;20:660–94. 17934078 10.1128/CMR.00023-07 PMC2176051

[11] NCBI Resource Coordinators . Database resources of the National Center for Biotechnology Information. Nucleic Acids Res. 2016;44:D7–19. 26615191 10.1093/nar/gkv1290 PMC4702911

[12] Madeira F , Park YM , Lee J , Buso N , Gur T , Madhusoodanan N , et al. The EMBL‐EBI search and sequence analysis tools APIs in 2019. Nucleic Acids Res. 2019;47:W636–41. 30976793 10.1093/nar/gkz268 PMC6602479

[13] Okonechnikov K , Golosova O , Fursov M . UGENE team Unipro UGENE: A unified bioinformatics toolkit. Bioinforma Oxf Engl. 2012;28:1166–7. 10.1093/bioinformatics/bts091 22368248

[14] Kerfeld CA , Scott KM . Using BLAST to teach “E‐value‐tionary” concepts. PLoS Biol. 2011;9:e1001014. 21304918 10.1371/journal.pbio.1001014 PMC3032543

[15] Altschul SF , Gish W , Miller W , Myers EW , Lipman DJ . Basic local alignment search tool. J Mol Biol. 1990;215:403–10. 2231712 10.1016/S0022-2836(05)80360-2

[16] Berman HM , Westbrook J , Feng Z , Gilliland G , Bhat TN , Weissig H , et al. The Protein Data Bank. Nucleic Acids Res. 2000;28:235–42. 10592235 10.1093/nar/28.1.235 PMC102472

[17] Pettersen EF , Goddard TD , Huang CC , Couch GS , Greenblatt DM , Meng EC , et al. UCSF Chimera—a visualization system for exploratory research and analysis. J Comput Chem. 2004;25:1605–12. 15264254 10.1002/jcc.20084

[18] Monto AS , DeJonge PM , Callear AP , Bazzi LA , Capriola SB , Malosh RE , et al. Coronavirus occurrence and transmission over 8 years in the HIVE cohort of households in Michigan. J Infect Dis. 2020;222:9–16. 32246136 10.1093/infdis/jiaa161 PMC7184402

[19] Zheng J . SARS‐CoV‐2: An emerging coronavirus that causes a global threat. Int J Biol Sci. 2020;16:1678–85. 32226285 10.7150/ijbs.45053 PMC7098030

[20] Prajapat M , Sarma P , Shekhar N , Avti P , Sinha S , Kaur H , et al. Drug targets for corona virus: A systematic review. Indian J Pharmacol. 2020;52:56–65. 32201449 10.4103/ijp.IJP_115_20 PMC7074424

[21] Debarnot C , Imbert I , Ferron F , Gluais L , Varlet I , Papageorgiou N , et al. Crystallization and diffraction analysis of the SARS coronavirus nsp10–nsp16 complex. Acta Crystallograph Sect F Struct Biol Cryst Commun. 2011;67:404–8. 10.1107/S1744309111002867 PMC3053173 21393853

[22] Decroly E , Debarnot C , Ferron F , Bouvet M , Coutard B , Imbert I , et al. Crystal structure and functional analysis of the SARS‐coronavirus RNA cap 2′‐O‐methyltransferase nsp10/nsp16 complex. PLoS Pathog. 2011;7:e1002059. 21637813 10.1371/journal.ppat.1002059 PMC3102710

[23] Wang Y , Sun Y , Wu A , Xu S , Pan R , Zeng C , et al. Coronavirus nsp10/nsp16 methyltransferase can be targeted by nsp10‐derived peptide in vitro and in vivo to reduce replication and pathogenesis. J Virol. 2015;89:8416–27. 26041293 10.1128/JVI.00948-15 PMC4524257

[24] Ke M , Chen Y , Wu A , Sun Y , Su C , Wu H , et al. Short peptides derived from the interaction domain of SARS coronavirus nonstructural protein nsp10 can suppress the 2′‐O‐methyltransferase activity of nsp10/nsp16 complex. Virus Res. 2012;167:322–8. 22659295 10.1016/j.virusres.2012.05.017 PMC7114426

[25] Khan RJ , Jha RK , Amera GM , Jain M , Singh E , Pathak A , et al. Targeting SARS‐CoV‐2: A systematic drug repurposing approach to identify promising inhibitors against 3C‐like proteinase and 2′‐O‐ribose methyltransferase. J Biomol Struct Dyn. 2020;1–14. 10.1080/07391102.2020.1753577 PMC7189412 32266873

[26] Waterhouse AM , Procter JB , Martin DMA , Clamp M , Barton GJ . Jalview version 2—a multiple sequence alignment editor and analysis workbench. Bioinf Oxf Engl. 2009;25:1189–91. 10.1093/bioinformatics/btp033 PMC2672624 19151095

[27] Corum J , Zimmer Bad C . News wrapped in protein: Inside the coronavirus. The New York Times. 2020 https://www.nytimes.com/interactive/2020/04/03/science/coronavirus‐genome‐bad‐news‐wrapped‐in‐protein.html.

[28] Chen Y , Liu Q , Guo D . Emerging coronaviruses: Genome structure, replication, and pathogenesis. J Med Virol. 2020;92:418–23. 31967327 10.1002/jmv.25681 PMC7167049

[29] Li F . Structure, function, and evolution of coronavirus spike proteins. Annu Rev Virol. 2016;3:237–61. 27578435 10.1146/annurev-virology-110615-042301 PMC5457962

[30] Fallon K , Bausch K , Noonan J , Huguenel E , Tamburini P . Role of aspartic proteases in disseminated *Candida albicans* infection in mice. Infect Immun. 1997;65:551–6. 9009312 10.1128/iai.65.2.551-556.1997 PMC176095

[31] Cutfield SM , Dodson EJ , Anderson BF , Moody PC , Marshall CJ , Sullivan PA , et al. The crystal structure of a major secreted aspartic proteinase from *Candida albicans* in complexes with two inhibitors. Struct Lond Engl. 1995;3:1261–71. 10.1016/s0969-2126(01)00261-1 8591036

