# Supplementary material for: Using COVID‐19 as a teaching tool in a time of remote learning: A workflow for bioinformatic approaches to identifying candidates for therapeutic and vaccine development
Source: Biochem Mol Biol Educ. 2020 Jul 29;48(5):492–8. doi: 10.1002/bmb.21413 (PMC7590178; doi:10.1002/bmb.21413)
Supplement: Supplementary file 1 — DATA S1 Supplemental materials 1 and 2 [file BMB-48-492-s001.zip › BMB_21413_Appendix_S1.doc]

**Supplemental Materials 1:**

Expanded Protocol:

For those who have less bioinformatics experience and/or encounter difficulties throughout the process we have developed the following more detailed protocol as a companion to our workflow. We recommend that instructors decide if they want to share this additional information based on a combination of students’ prior bioinformatics experience and the time allotted for the project.

1) Download sequences from NCBI. Students should document the unique accession number associated with the genome, and also make sure that they are only downloading amino acid sequences. If multiple proteins will be aligned simultaneously, concatenate their sequences first in a text file.

2) Paste sequences or upload a file into the [Clustal Omega Multiple Sequence Alignment (MSA) online tool](https://www.ebi.ac.uk/Tools/msa/clustalo/) and submit the job.

3) Open alignment results file in Unipro UGENE. A tutorial on analyzing multiple alignments in UGENE can be found at <http://ugene.net/multiple-sequence-alignment-overview/>.

4) Under the highlighting tab, color the alignment by *Percentage identity*, highlight by *Conservation level*, and choose an appropriate conservation threshold. The red boxes and arrow below indicate which settings students should adjust to perform these actions.

5) UGENE will identify conserved polypeptide sequences that satisfy the conservation threshold. The red box below highlights what a region that is strongly conserved will look like. If students are encountering difficulty identifying highly conserved regions, they may find it beneficial to manipulate the thresholds.

6) BLAST the candidates against human proteins (BLASTp) to check for a potential autoimmune response or toxic side effects of a therapeutic. The red box and arrow denote where to find the E value within BLAST results. Note that the large E value shows this ‘match’ to a human protein is not meaningful, suggesting there will be no cross-reactivity. Mini courses on using BLAST can be found at <https://www.ncbi.nlm.nih.gov/Class/minicourses/>.

7) Search for the protein structure in [RCSB Protein Data Bank](https://www.rcsb.org/) using a sequence from the conserved region of the protein being visualized. In the example below, the SARS-CoV-2 sequence from the conserved region was used.

8) Identify the correct protein structure via PDB ID (red box/arrow). If students encounter difficulty identifying the correct protein structure in the PDB, they are encouraged to perform a literature search for publications detailing the crystal structure; these publications will include a PDB ID to locate the protein.

9) Open [UCSF Chimera](https://www.cgl.ucsf.edu/chimera/) and fetch the protein structure by the PDB ID

10) Select and color the previously identified conserved sequence(s). Tutorials on using Chimera can be found at <https://www.cgl.ucsf.edu/chimera/tutorials.html>.
