# Supplementary material for: Using COVID‐19 as a teaching tool in a time of remote learning: A workflow for bioinformatic approaches to identifying candidates for therapeutic and vaccine development
Source: Biochem Mol Biol Educ. 2020 Jul 29;48(5):492–8. doi: 10.1002/bmb.21413 (PMC7590178; doi:10.1002/bmb.21413)
Supplement: Supplementary file 1 — DATA S1 Supplemental materials 1 and 2 [file BMB-48-492-s001.zip › BMB_21413_Appendix_S2.doc]

**Supplemental Materials 2:**

Additional result to demonstrate versatility of the workflow:

Although our workflow was designed with COVID-19 in-mind, we want to further emphasize the versatility of this approach, making it useable for many other therapeutic and vaccine inquiry-based exercises. Here we briefly demonstrate using this workflow to develop a vaccine candidate sequence from the Sap proteins of the pathogenic fungus *C. albicans*. Saps are a family of 10 secreted aspartyl proteases that have long been recognized as important *C. albicans* virulence factors [30].

We aligned the amino acid sequences of all 8 secreted Saps with the three human closest homologs (cathepsin B, pepsin A and renin), identifying 5 regions that are conserved within all *C. albicans* Saps (Figure S1). We noticed that Region #4 is highly conserved within *C. albicans* Saps and simultaneously highly divergent to human homologs. This region is a stretch of 35 amino acids with the consensus sequence EATGTPYDNVPVTLKKQGIIAKNAYSLYLNSPEAS. Notably, while Saps are not generally present in humans, region #4 is not present anywhere in the human proteome, ensuring that cross reactivity is not a concern. When we looked for the location of this sequence on the available Sap2p crystal structure [31] we found that it lies on a highly exposed region of the protein that is ideal for potential antibody binding (Figure S1, top left corner, yellow loop). While this example is brief, we hope that by including it we have shown that our workflow can be adjusted successfully to study diverse pathogens.

Figure S1: The five conserved domains identified by the workflow, which are reported in yellow. Domains 3 and 5 contain the two catalytic subunits (highlighted in red) while domain 4 is the domain proposed for potential vaccine development. The top left corner represents the 3-D structure of Sap2 in which domain 4 has been highlighted in yellow. The structure was generated in UCSF Chimera, the conservation map was generated in Jalview, and highlighted regions in UniproUGENE.
